# Physical rehabilitation in the context of a landslide that occurred in Brazil

**DOI:** 10.1186/s12889-019-7964-x

**Published:** 2019-12-02

**Authors:** M. L. Carvalho, C. M. Freitas, E. S. Miranda

**Affiliations:** 1 Instituto Federal de Educação, Ciência e Tecnologia do Rio de Janeiro, Rua Carlos Wenceslau, 343, Realengo, Rio de Janeiro, RJ 21715-000 Brazil; 2 Centro de Estudos e Pesquisas em Emergências e Desastres em Saúde, Escola Nacional de Saúde Pública, Fundação Oswaldo Cruz, Rua Leopoldo Bulhões, 1480, Manguinhos, Rio de Janeiro, RJ 21041-210 Brazil; 3 Programa de Pós-graduação em Administração e Gestão da Assistência Farmacêutica, Faculdade de Farmácia, Universidade Federal Fluminense, Rua Mário Viana, 523, 867, Niterói, RJ 24241-000 Brazil

**Keywords:** Natural disasters, Health service delivery, Health access to health services, Physical rehabilitation

## Abstract

**Backgrounds:**

The efforts to develop research and training on physical rehabilitation in regards to disasters is considered recent worldwide. In the late evening of the 11th up until the 12th of January of 2011, the most massive natural disaster occurred in Brazil with extremely heavy downpour, abrupt flood, as well as landslides on multiple areas of the Mountain Region of Rio de Janeiro. The objective of this research was to investigate the challenges in terms of physical rehabilitation provided by this event

**Methods:**

The cross-sectional mixed method’s study, which was conducted in the city of Nova Friburgo, used two different data sources: hospital records on traumatic injuries pre and post disaster, and interviews with key informants - victims who suffered injuries related to the disaster, professionals from rehabilitation services in the municipality, and also the city’s health service management. Pearson’s chi-squared test was performed to evaluate statistical significance between the week of a given incident and the type of injury. Interviews were transcribed and analysed through content analysis.

**Results:**

A total of 2326 hospital records and 27 interviews were analysed. The proportion of traumatic injury in the municipal emergency service increased from 16% in the prior week, to 40% in the week post-disaster (*p* <  0.0001). Different injuries were identified: multiple fractures, crushing, amputation, perforation of soft tissues, inhalation of dust and establishment of chronic conditions through stress. Despite this scenario, out of the 16 health professionals interviewed, twelve did not observe an increase in the demand for outpatient rehabilitation services after the disaster. Interviews with the victims revealed that the pathways for care ran into different barriers. From 11 victims interviewed, only one received complete physiotherapy care through the public health services in the city, while all others hired additional assistance, received volunteer services, had assistance in other cities or remained without rehabilitation.

**Conclusions:**

The needs for rehabilitation increased after the disaster; however, the demand was repressed due to different barriers such as competing needs and possible lack of medical referral. Recommendations were made, including the action of performing a search of victims with rehabilitation needs.

## Backgrounds

The efforts to develop research and training on physical rehabilitation in disasters is considered recent worldwide and is recognized as an emerging speciality within the field of Physical Medicine and Rehabilitation. An important milestone in this context was the first *Rehabilitation Disaster Relief* Symposium, which occurred in 2011. At that time, it was evidenced that the response plans and protocols for acute care in disasters usually did not include interventions of rehabilitation, resulting in negligence of immediate rehabilitation needs and the establishment of medical complications and permanent disabilities which could certainly be avoided [1]. Since 2011, international parameters for rehabilitation assistance in disasters were established with the purpose of preventing impairments and associated disabilities [2].

Concerning the rehabilitation teams, they should be present in the disaster from the initial response stages and must be part of the medical emergency teams of type 1, 2 or 3. Type 1 team must be capable of providing basic rehabilitation assistance, or refer patients to rehabilitation services of higher complexity. Types 2 and 3 must be capable of offering autonomous rehabilitation services, including care in situations consisting of exposed fractures, peripheral nerve injuries, amputations, burns. It must also be able to offer respiratory care, psychosocial support, and daily activity training. Types 2 and 3 teams must have at least one rehabilitation professional for every 20 beds. In relation to the professions involved, they should be multidisciplinary, composed of at least one physiotherapist, one occupational therapist, one physiatrist and/or rehabilitation nurse. Other aspects which must be established would be: Equipment, minimum physical structure, as well as considerations regarding the handling of particular situations (for example: spinal cord injury, head traumas, and people with previous physical impairments), as well as the rehabilitation performance in outbreaks of transmittable diseases [3].

Besides the wounds, which may be deep and reach soft tissues such as nerves, muscles, and tendons, the traumas occurring in natural disaster may cause dislocation, crushing, amputation or burns, all of which demand the action of rehabilitation services [1].

As for the epidemiology of lesions specifically in landslides, literature is sparse [4]. Severe injuries, such as compression and crushing of the pelvis and thorax, as well as traumatic asphyxia [5] are regularly mentioned. A study compared flood and landslide injuries in Uganda and showed a higher lethality rate for landslides and higher injury rates in the population affected by the flood. On the other hand, fractures and lacerations were more common in landslides [6].

Additionally, due to stress and the impact on the health system, chronic patients may suffer an exacerbation of their symptoms, destabilising their condition. Thus, disasters compromise the treatment of chronic diseases and ongoing physical rehabilitation processes as much as they bring about new rehabilitation demands [7, 8].

In Brazil, rehabilitation is foreseen to occur in all levels of care through a public system of universal access (Unified Health System - SUS). The primary care, a direct attribution of the municipal level, must perform an active search of the rehabilitation needs, offer longitudinal care considering the family and the community, and refer the same to services of higher complexity when necessary [9].

At the dawn of the 11th to the 12th of January, 2011, the Mountain Region of Rio de Janeiro was the background of the biggest disaster registered in Brazil in terms of immediate deaths (*N* = 918), leaving 45 thousand people homeless or displaced [10]. The disaster combined an abrupt flood with multiple points of landslides after a few hours of heavy rain. Many people were injured, therefore a need for the establishment of field hospitals arose [11]. Traumas with bruising and injuries on the skin and extremities were the most common occurrences among the survivors [12]. Public infrastructure was largely affected, including health services. Seventy-three bridges were destroyed and the main access routes to the region, as well as the energy, communication, and water supply were interrupted either totally or partially. It is estimated that agriculture and commerce had economic losses of about 145 and 253 million dollars respectively. The city of Nova Friburgo was the most affected in terms of immediate deaths (*N* = 429) among the 16 municipalities that form this Mountain Region [13]. Despite the disaster having occurred in 2011, there are no studies of post-disaster physical rehabilitation in Brazil.

The purpose of this research was to investigate the challenges in terms of physical rehabilitation provided by the disaster in 2011. The initial hypothesis was that there was an increase in the number of people with traumatic injuries after the disaster, leading to a visible increase in rehabilitation needs and demands. The research was achieved through the analysis of needs related to rehabilitation from different cases of disaster victims, as well as the response of the healthcare system to physical rehabilitation needs from the point of view of health professionals and the local health service manager.

## Methods

**Fig. 1:**
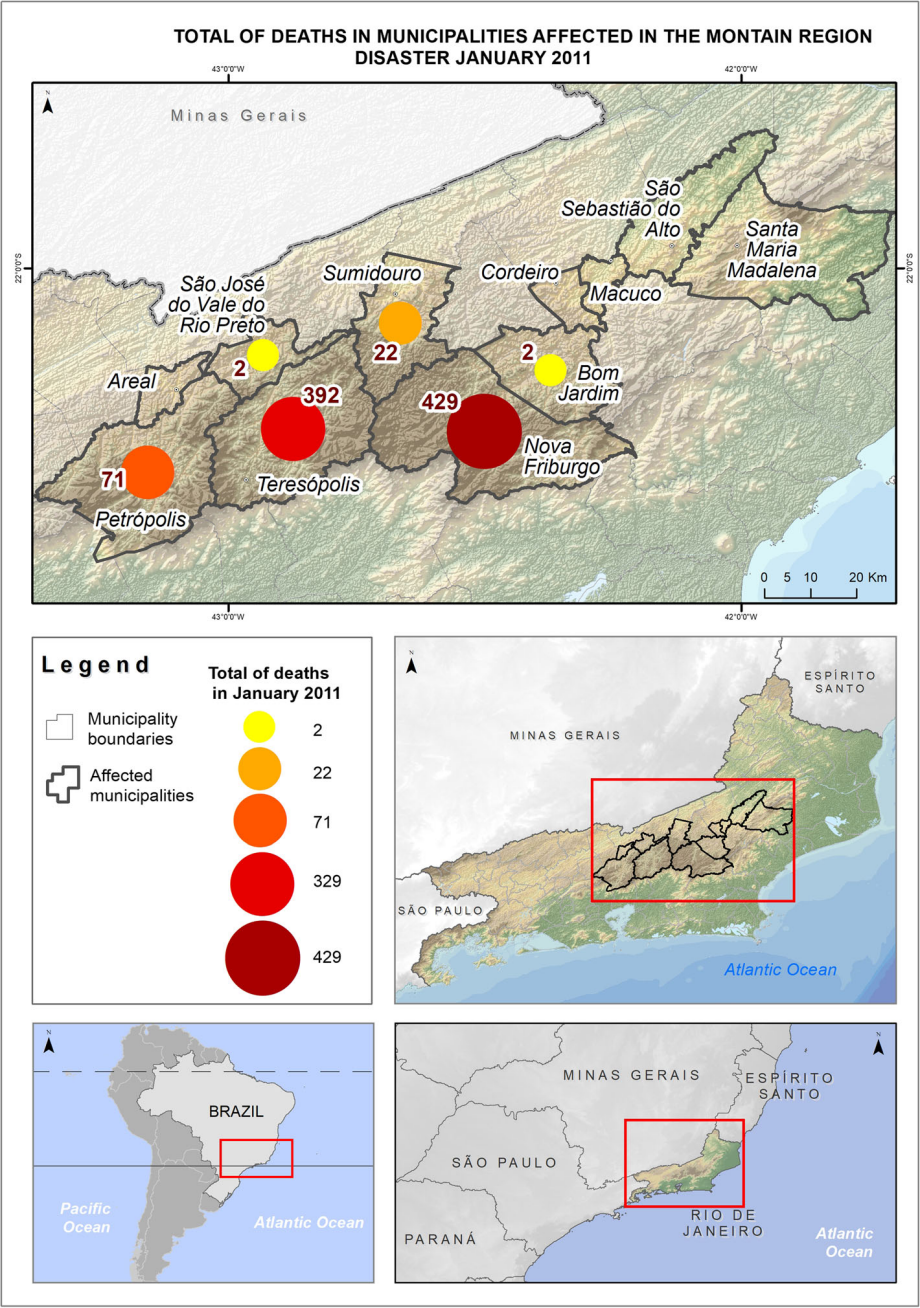
Total of deaths in municipalities affected in the montain region disaster January 2011

A cross-sectional mixed methods study was conducted. It was used as a quantitative investigation of the number of people with traumatic injuries who arrived at the municipal hospital the week before and after the disaster, with a qualitative study through interviews with key informants in the city of Nova Friburgo (Fig. 1).

The increase in the number of people with traumatic injuries at the municipal hospital would point to an increase of rehabilitation needs [1]. In general, interviews with professionals, managers and victims could show whether it really was what had happened and how it happened. Specifically, the interviews with the victims should qualify the needs of rehabilitation and show the real paths of the search for care. And interviews with health professionals and management would show how these needs had been translated into demand for the health system. It is a convergent mixed method’s design [14, 15] with quantitative data pointing to general trends, while qualitative data provide more detailed information from the perspective of individuals.

### Hospital records

In order to detect the changes in the patterns of health problems and a possible proportional increase in the needs for rehabilitation, given that the traumatic injuries should cause this increase, patient registries from the emergency department visits on the public hospital in the municipality were consulted. These registries were in relation to a week before and after the disaster, in which traumatic injury and other health problems were identified and organized into a Microsoft Office Excel® software version 2013 database. These were considered as traumatic injuries: direct trauma, traumatic wound and animal bites.

### Interviews with key informants

Three interest groups were defined: victims which suffered direct injuries related to the disaster, professionals acting in rehabilitation services in the municipality, and municipality’s health management. Among the victims, those who suffered injuries to muscles, tendons, nerves, bones or viscera,as well as cases in which the disaster had impaired health generating the need for rehabilitation were included. Those who suffered only skin lesions without motor repercussions were excluded. The interviews were conducted in the local language, Portuguese, from the period between September 15th, 2015 to January 15th, 2016.

The victims were identified by means of a combination of sources, such as hospital records, neighbourhood associations, charity institutions, professionals from rehabilitation services, and the indication of the affected people themselves. The number of interviewed victims was defined when data saturation was reached [16]. For this research, it was considered satisfactory to find either victims who had assistance through public, private health service (PHS) or long-term volunteer services, or no assistance.

The selection of health professionals started with a search on the National Registry of Health Establishments (Cadastro Nacional dos Estabelecimentos de Saúde - CNES) of those establishments that offer specialized public rehabilitation services. These are physiotherapy services; orthotic, prosthetic and special materials for rehabilitation; as well as rehabilitation services. Physiotherapists from the PHS were selected for the interview given their volunteer participation in the disaster’s recovery process. The victims or other professionals had been referenced the same. Finally, the municipal manager of rehabilitation/physiotherapy outpatient care was also invited to participate in the research.

The interviews were performed by means of semi-structured instruments, developed for each of the different groups involved. The interview with the victims emphasized the point of view and experience of the impacted population, their rehabilitation needs in consequence of the disaster and their possibility of access to the healthcare services necessary for their recovery. The interview with professionals, however, was to understand their point of view about the way that the disaster interfered or what was demanded of rehabilitation services, in what manner the rehabilitation services responded to the disaster, and in what manner the response could be more efficient. Concerning the management, the instrument directed to identify if there was an increase in the perceived demand and what the response strategies were.

### Data analysis

The data about the types of assistance performed at the public Hospital were collected from emergency reports. From the number of traumatic injury and other health problems, in the weeks prior and post disaster, it was possible to calculate the proportions before and after the event. The statistical analysis software R (version 3.5.2) was used to apply Pearson’s chi-squared test and the evaluation of statistical significance to avoid the possibility of a difference in the pattern found after the disaster had occurred by chance.

The analysis of the material collected in the interviews was performed through the proposition of a thematic analysis modality of content analysis [17]. Consequently, the transcription stages of the material collected in the interviews were performed, as well as a comprehensive and exhaustive reading of the entire material, the preparation of the initial postulates, selection and gathering of analysis units according to these postulates, identification of meaning cores in the grouping of themes into categories.

## Results

A total of 2326 hospital records and 27 interviews were analysed. The people interviewed included 11 victims, 16 health professionals (including 14 physical therapists, one occupational therapist and one social worker) and a manager of municipal rehabilitation services.

As it was an event that mobilised the people of the municipality emotionally, all victims consulted that met the inclusion criteria wanted to participate, even those who developed some emotional trauma. As for health professionals, only one from an outpatient clinic that provides physiotherapy services to the city did not want to participate. Among the victims interviewed, eight were women and three men, with ages ranging between 20’s- 30’s and 60’s- 70’s.

The health problems communicated in the emergency records of Nova Friburgo municipal hospital in the week before and after the disaster revealed an increase in the proportion of traumatic injury a week after the event. On the previous week, the percentage of traumatic injury was 16 and 84% for other health problems. On the following week, the number had risen to 40% of traumatic injury and 60% for other health problems. Based on the Pearson’s chi-squared test, it was possible to reject the null hypothesis of independence between the variables “the week before and after” and “traumatic injury and other health problems, allowing the assumption of statistically meaningful association (*p*-value < 0.0001) between them (Table 1).

**Table 1:** Traumatic injury and other health problems at the emergency of the municipal hospital, the week before and after the disaster

Hospital records	Week before	Week after	*P*-value
Traumatic injury	303 16%	213 40%	< 0.0001
Other health problems	1497 84%	313 60%	< 0.0001
Total	1800 100%	526,100%	

To the left, the absolute number, and to the right in percentage. *P*-value obtained through Pearson’s chi-squared test

Among traumatic injury, direct physical trauma, whether accidental or intentional, traumatic wounds and animal bites were reported. Concerning other health problems, fever, hypertensive crisis, diarrhoea, nausea, vomit, intoxication, visceral pain, weakness, faintness, suspicion of urinary infection, earache, sore throat, headache, chest pain, coughing, bug bites and records of pain and/or loss of musculoskeletal function without history of trauma were identified. All complaints from psychological origins such as anxiety, agitation, crying, depression, behavioural disorders, and stress also counted as other health problems.

Regarding the interviews, the categories that emerged during the analysis process were: 1) types of injuries caused by disaster and the consequences for the individual’s health and life; 2) the paths disaster victims had to go through in search of physical rehabilitation; and 3) supply and demand for post-disaster rehabilitation services.

### Types of injury caused by the disaster and the consequences for health and life

Eleven victims of disaster were interviewed. The interviews with victims aided in the comprehension of the injury mechanisms that had appeared because of the disaster.

Out of the eleven victims interviewed in this research, seven suffered fractures, five had multiple fractures and two only had upper body fractures. One suffered crushing and consequential amputation of one of the lower limbs. One suffered an injury in the soft tissues and a rib fissure. One inhaled dust, worsening a pre-existing condition of respiratory allergy and two developed chronic pains related to stress (Table 2).

**Table 2:** Characteristics of interviewed victims, types of injury and the consequences for health and life

Victim	Sex	Age	Physical injury and injury mechanism	Private healthcare plan	Received rehabilitation assistance	Had a job before the disaster in 2011	Had a job in 2015
Case 1	F	40’s - 50’s	The house collapsed but she was not buried. Inhaled a lot of dust at the moment of the landslides.	No	No	Yes	No
Case 2	F	40’s- 50’s	The house collapsed and her body was dragged. Suffered a fracture on the forearm and an open fracture on the leg.	Yes	Yes	Yes	No
Case 3	F	40’s- 50’s	The house collapsed. She was buried, fractured the left arm and amputated the right leg.	No	Yes	Yes	No
Case 4	F	60’s- 70’s	Did not suffer direct physical injury. Chronic pains aggravated by stress after the disaster.	No ^a^	Yes	No	No
Case 5	F	50’s- 60’s	The house collapsed. She was buried and fractured the right arm in two points.	No	Yes	Yes	Yes
Case 6	F	30’s- 40’s	The house collapsed. She was buried, suffered a fracture on the pelvis and femur.	No	Yes	Yes	Yes
Case 7	F	60’s- 70’s	The house collapsed. She was stuck under a slab and managed to get out by herself. Dislocated the clavicle in the effort.	No	Yes	No	No
Case 8	F	20’s- 30’s	The house collapsed. She was buried and suffered fractures on both feet.	No	Yes	Yes	No
Case 9	M	40’s- 50’s	Did not suffer direct physical injury. Chronic pains and symptoms such as systemic hypertension, irritable bowel syndrome and gastritis manifested after the disaster.	No ^a^	Yes	Yes	Yes
Case 10	M	30’s- 40’s	Was buried up to his chest. Suffered excoriations, muscle hernia on the left leg, fissured the rib and sprained an ankle.	No ^a^	Yes	Yes	Yes
Case 11	M	50’s- 60’s	Fell in a hole on the street and suffered a fracture on the right forearm.	No	Yes	Yes	No

Source: Designed by the author from the data collected on fieldwork. ^a^Hired later

The direct injury mechanisms involved burials, being dragged by landslides, water, debris, crushing of some part of the body, falling into open holes in the street, trying to escape a restrictive location and staying too long in a dusty environment. The most common injuries were skin excoriations. However, these did not generally cause motor sequelae, while fractures, crushing and deep perforations in soft tissues were the main injuries that caused motor sequelae (Table 2).

In relation to the individuals that developed chronic pain due to stress, one of them, a 40’s – 50’s year-old man, was directly involved with the demands of his district, which was heavily affected by the disaster. According to him, the fight for improvements and the district’s recovery made him relive the traumatic situation countless times, somatising it in the form of systemic hypertension, back pains, stomach ulcer, gastritis, reflux, aside from irritable bowel syndrome for about 4 months.

Another case of health deterioration due to stress after the disaster was of a 60’s − 70’s year-old woman. After having her home interdicted because it was under the risk of collapsing, she moved to the residential condominium built specifically to house victims of different communities affected by the disaster. This changed her routine completely and interfered especially in her relationship with individuals from her neighbourhood. She felt invaded and threatened in her values due to the relationship with families with social habits completely different from her, and this contributed to the establishment of a chronic and debilitating low back pain condition.

Concerning their employability, out of the eight female victims interviewed, six had jobs before the disaster. By the time of the interview, two of them had returned to their jobs, one was looking for a job, and three considered themselves unable to work. They argued that their education was insufficient and their work would depend directly on the bodies as they would search for manual labour. Among the three men interviewed, two had already returned to the job market and one was looking for a job, despite reporting physical limitations.

### Paths taken by victims of the disaster in search of physical rehabilitation

The search for physical rehabilitation after the disaster in Nova Friburgo consisted of many diverse and non-linear paths, leading to experiences with interruptions, fresh starts and discontinuations; amid public, private and nonprofit services; at home, in the municipality or at far away reference centres (Table 3).

**Table 3:** Organizations involved in rehabilitation assistance used by the victims interviewed

Organizations involved in rehabilitation assistance	Number of victims
Municipal public outpatient clinic	3^a^
Public Rehabilitation center in another municipality	2
Private outpatient clinic	4
Private clinic or home - voluntary service	3
Not sought or referred to rehabilitation care	1

^a^two victims received treatment by the municipal public service and, later, voluntary service

Long-term volunteer assistance was the one offered freely by professionals not only in the acute phase of the disaster, but as long as there was a need. Among the 11 interviewed victims, three used long-term volunteer work for physiotherapy services. All of them received full assistance, but the longest treatment took about 2 years, this being until the patient could walk again.

Five received rehabilitation assistance in public institutions. Two were taken to a hospital 104 Km away from their hometown after the rescue, where surgery was performed on them and they received hospital rehabilitation assistance. Only one was able to continue the treatment there. The other individual received volunteer physiotherapy assistance at home, offered by a former neighbour who was a physiotherapist. Yet, among the three victims accepted by the public health service of Nova Friburgo, only one received full physiotherapy assistance. One individual received ten physical sessions and was discharged before recovery, continuing at home on her own account, without orientation, according to her own intuition. The last one had given up after a few sessions in which she attended for 5 minutes, later searched and received volunteer assistance in a PHS (Table 3).

Concerning the victim that continued the treatment in the rehabilitation centre, despite going to a reference centre and having acquired a state-of-the-art prosthesis, according to the patient, the 104 km distance from her home to the rehabilitation centre, as well as the physiotherapist’s demands for her to adapt to the prosthesis, demotivated her attendance to the assistance and recovery process. This patient was retired on disability, is partially dependent on instrumental daily activities and presented depressive and anxiety symptoms related to the disaster trauma which has damaged her well-being.

“*The transport... the car was already scheduled by the time the week had come up [that is why I wouldn’t go every week](...) I was under a lot of pressure, the physiotherapist that took care of me said that my prosthesis was the best one and that I had to try hard. What made it difficult was my financial situation, pressure from my physiotherapist and my evaluations, which were bimonthly”.*

On the other hand, the victim who received long-term volunteer assistance at home had fewer technological resources but did in fact receive regular service, incentive up to a certain point and respect for her needs, not only physical but also social, related to child care. The patient regained her stride, was able to go back to her job and became independent for the instrumental daily activities with few adaptations.

“So*, when I started walking, I couldn’t even bend my leg. After therapy. I was satisfied because I had to get my son’s life back on track, buying everything for him again, giving him more attention, because he had lost his father, right? So I left physiotherapy as fast as I could because I only wanted to walk, right? If I could bend the leg, for me that was great!”*

Only one victim had a private healthcare plan at the time of the disaster and received complete physiotherapy treatment through the plan. Three victims hired healthcare plans after the disaster. One of them affirms having hired it to obtain adequate treatment for chronic back pain, and was undergoing treatment at the time of the interview. The other relates having given up on physiotherapy through the healthcare private plan because the expected results were not achieved. Only one victim paid directly for physiotherapy, the same victim who recovered completely.

Only one victim affirmed not searching for rehabilitation physiotherapy services. She had her respiratory system affected the most and was unaware of the existence of pulmonary rehabilitation, as she was not referred by the health services towards this treatment option.

### Supply and demand for outpatients rehabilitation services after the disaster

For the outline of rehabilitation services supply after the disaster, descriptions of services rendered were brought from health professionals responsible for such services in the public sphere (a social worker, an occupational therapist and eight physiotherapists), a sample of health professionals from the PHS (six physiotherapists that provided long-term volunteer assistance after the disaster), and a manager of the public rehabilitation service in the municipality of Nova Friburgo. The perspective concerning demand was composed of explanations brought by health professionals and the manager, as well as the victims themselves.

Out of the 16 interviewed professionals, twelve did not observe an increase in the demand for rehabilitation services after the disaster. The increase was also not perceived by municipality interviewee management of rehabilitation services.

*“I thought, there is going to be a huge increase after the tragedy, and there wasn’t anything like that.”* (physiotherapist, community health centre).

*“Here in the health centre there was no increase, in other places I don’t know, I don’t have access to those.”* (physiotherapist, community health centre).

*“Nothing came up that would show an increase like this, that would draw so much attention from the professional physiotherapist that could make it reach the management as a demand.”* (manager of public rehabilitation services).

Of the professionals that did not identify an increase of demand for rehabilitation services, four answered on behalf of private institutions, two for hospitals that do not offer outpatient rehabilitation services, two for non-profit organizations that do not serve the general public, only children with developmental delay and four by municipal services.

What was most evident was the reduction of ongoing treatments, because of either the decrease in demand or situations that impaired the supply. Patients of PHS claimed difficulties of transportation to get to the clinic, financial losses, need to support someone in the family, or other priorities arising, such as the recovery of documents, belongings, and reorganizing their personal life. The three non-profit organizations were in recess due to vacation period when the disaster struck, as they work as a school and this being the reason for the vacation period. Two of these had their structures affected and the renovations took months to conclude.

Besides, the emotional shock and difficulty to transit through the city worsened the access and treatment continuity after their reopening. Residents of the rural area had even more trouble to commute. This situation took about 6 months to normalize itself. Professionals from two hospitals reported that the structure of these services was halted totally or partially, and because of that, they needed to transfer patients for care to other health clinics. In one of these services, the outpatient physiotherapy service space was permanently closed in 2007 after another flood destroyed the facilities.

Only three professionals observed an increase of demand for rehabilitation services in direct consequence to the disaster. This happened in a non-profit association that assists the public and children with developmental delay, in a PHS and in only one health clinic, located in a district that was particularly affected by the disaster. In two of these cases, the increase of demand faced the structural damages which occurred due to the disaster:

*“Here we usually treat the neurological patients. After the disaster, we started having a lot of the orthopaedics part too. Something that did not happen before the disaster (...) We stopped working at the time because we were affected a bit here. Then we went somewhere else, to a court. It was very hard to work in my sector. We had to adapt gurneys, the location, devices that we did not have, and this disrupted us a lot at the time.”* (physiotherapist, non-profit organization).

*“The demand increased a lot due to the people that suffered fractures, sprains and were directly traumatized by the tragedy. In quality, the structure continued the same. Actually, it got worse. Because when the tragedy occurred at daybreak, people who wanted to rescue... the places to rescue were nearby... and people forced the doors to steal – no, not to steal – to get the gurneys. So I had no gurneys to work.”* (physiotherapist, health clinic).

In two PHS, the increase in the demand for rehabilitation by indirect consequences of the disaster was observed, as well as injuries caused by the cleaning and reorganisation of physical spaces, or even by mental stress:

*The cleaning and tidying up of everything that was left also caused a lot of problems for many people, physical problems, injuries to the tendons*...” (physiotherapist, PHS).

*“Then, concerning the rehabilitation process... This disaster created a lot of psychological stress, right? Most have developed some trauma, it starts to rain and you see people panic. So what did the psychological change that occurred in the population generate? Back pain, headache, so... they somatised it. So the demand increased... Some patients showed up with fibromyalgia... all diseases of emotional background.”* (physiotherapist, PHS).

When the increase in the demand was observed, it did not happen immediately after the disaster, but about two to 3 months later. The interviews with victims confirmed and explained this delay between the disaster and search for outpatient physiotherapy services. Before searching for these services, patients had to go through hospitalizations, surgeries and immobilization periods. Additionally, the emotional shock, other urgent needs and, in some cases, the need to aid some other family member even more injured. The speech below illustrates well the situation of the victim that suffered an injury on the upper limbs, needed outpatient rehabilitation services, but postponed this search to take care of his family due to the death of his son-in-law, the daughter gravely injured and a young grandson:

*“He [the doctor] said that when things calmed down, I should look for an orthopaedist. However, I couldn’t leave my grandson because he couldn’t stay with his dad, so I ended up doing everything (...) What they would do to me was going to interfere with this. I had to look after my grandson, I had to take care of my daughter’s life, she was hospitalized in Rio.”*

The interview with management reveals that the city never had an investment in the rehabilitation sector. The city hall has only some agreements with physiotherapy clinics.

*“We always worked with agreements, with clinics that weren’t rehabilitation clinics, but offered some service to this area, such as physiotherapy. Anyway, something also with the agreement (...) for some situations, but actually the service was never organized, never had a municipal rehabilitation centre.”*

It is possible to affirm that municipality management recognised that the municipality’s rehabilitation structure was precarious.

After the disaster, the rehabilitation needs did not come as a problem to the management, which acknowledges not having performed an active search because problems of immediate response, disease control, and psychosocial demands were prioritized.

*“...we ignored this possibility with the issue of rehabilitation in relation to the disaster because we worried a lot more about the emotional trauma and diseases arising from it, such as diabetes, hypertension, than physical injuries. Somehow, we created an idea that the disaster killed, but did not leave sequelae. It was possibly a very erroneous evaluation of the health service.”*

Knowing the fragility of the municipality’s physiotherapy public services and having noticed the increase in the need for physiotherapy services, six physiotherapists acting in the PHS offered long-term volunteer physiotherapy service. They communicated the offer of free assistance in their private clinics through personal referrals, at institutions (such as churches), or among medical colleagues. They were expecting a higher demand than what they effectively received. In this sense, there were individual, as well as collective initiatives. A group of about five physiotherapists, with clinics in different districts of the city organized themselves in a network to offer these services to victims of the disaster. According to the interview, a proposal was sent to city hall, but they received no response by the time of the field research. Interview with the management reveals that this proposal never reached its destination.

## Discussion

This study showed that there was a proportional increase in traumatic injuries at the Nova Friburgo public hospital after the disaster as is expected in extreme weather events [18], resulting in physical, permanent, or temporary impairments [19] and a growth in the rehabilitation needs. On the other hand, the majority of rehabilitation services didn’t identify a surge in demand. The interviews with the victims and health professionals pointed out that the demand existed, but was repressed for competing needs and lack of access and knowledge of the rehabilitation service.

It is worth observing that there was no increase in the gross number of service cases of traumatic injuries at the only municipal hospital in Nova Friburgo on the week after in relation to the week before the disaster. It is also important to mention that the absolute numbers of traumatic injury and other health problems had declined in the week following the disaster due to the interdiction on part of the hospital. However, by means of victim reports, it was possible to infer that the assistance occurred at other emergencies, field hospitals or even at other municipalities.

This is a pioneer study about physical rehabilitation in situations of natural disasters in Brazil. Most international studies about rehabilitation after natural disasters were performed for earthquakes. In these cases, there is often the occurrence of spinal cord injuries, which was not observed in Nova Friburgo. If there was any case, it did not reach the municipality’s public rehabilitation for treatment or monitoring. Nonetheless, injuries such as crushing, long bones fractures, pelvic fractures, multiple fractures, chest injury, strains, and infectious wounds, normally related to earthquakes [20, 21], also occurred in Nova Friburgo.

The study of Pereira et al. (2013) [12] shows that the most common injuries in the disaster of the Mountain Region were wounds that needed only asepsis, debridement and suture. Deeper fractures, strains, and injuries found in soft tissues were less common among the survivors. Perhaps this fact has distracted the attention of health professionals and managers in relation to rehabilitation needs.

Out of the 11 interviewed victims, three lost capacity to work due to the disaster. In none of these cases, the total loss of capacity to perform the instrumental activities of daily life was observed. Besides being all women, what the three cases have in common is that the loss of capacity for work is not related only to the loss of motor function, but mainly with low educational level, which complicated the aid of possible mechanisms of adaption to other activities. These examples reinforce the importance of understanding rehabilitation beyond the biomedical aspects to reach psychosocial, economic and behavioural dimensions. It is clear within this small sample that to these women, rehabilitation was an incomplete and interrupted process. As Ferdiana et al. (2014) [22] point out that cases in which the individual performed work activity with great physical demand before a spinal cord injury require an additional investment in education and vocational training to allow reintegration on the labour market.

Two interviewed victims (one male and one female) reported the worsening of chronic pain after the disaster, but not because of direct injury. Instead, it was attributed to the mental suffering directly related to the disaster. Depression and anxiety symptoms commonly occur after the experience of a traumatic event and these are related to chronic pain in the musculoskeletal system [23], as Yabuki et al. (2015) [24] found among people displaced in Fukushima after the disaster in March of 2011.

In different contexts, chronic pains may entail situations of physical disability, even making the individual look for emergency medical assistance due to great pain [25]. Many times, when realizing that there is a limitation of activities or unbearable pain, the cause is already instilled. In case of disasters, it is possible to include economic losses, disorganisation of life routine and new demands that arise daily, to what make affected individuals delay the request for treatment, and when they do, there is no direct association with the disaster. Thus, some health needs generated by the disaster are lost on everyday issues.

The inconvenience of a symptom diluted in everyday health problems is that once it manifests, the extra resources offered to the municipality to respond to the needs that have arisen due to the disaster, many times are spent, The assistance to chronic pain and physical disabilities demanded a specific rehabilitation care system that did not exist previously in the studied municipality and was not established after the disaster.

Out of the 11 interviewed victims, all of them needed rehabilitation services after the disaster. However, their paths to obtaining the treatment was varied. The rehabilitation service through SUS in the city assisted only three of these cases, and only one reported the treatment as successful. The two other cases were interrupted. On one of the cases, the interruption happened after 10 sessions without full recovery. On the other, the patient had given up due to the precariousness of the assistance. These findings reveal the situation of fragility that the public rehabilitation services in the municipality undergoes.

The prescription of 10 physiotherapy sessions is a custom among physicians when referring the patient for rehabilitation, mostly when the physiotherapy sessions are paid by private healthcare plans or by SUS, which determine rules for the use of services. It is a matter of managing the distribution of physiotherapy services that does not articulate with the real needs of the patients. The number of physiotherapy sessions cannot be defined because it depends on a series of factors that interfere with the functional recovery, including location, nature, extension of the injury, the patient’s age, clinical condition, and daily routine, as well as the level of education [26, 27].

The PHS was responsible for the treatment of seven of the eleven interviewed victims. Among them, only one abandoned the treatment (offered by private healthcare plan) because improvement was not observed. Another was still undergoing treatment in the interview period. The other five interviewees assisted by other means, had success in relation to their functional recovery. Three of them were serviced by long-term volunteer assistance. In Brazil, the PHS should work to complement SUS (Law 8.080/90). In practice, however, the contrary may occur, as observed in this sample.

Two victims with severe injuries were hospitalized in a national reference Traumatology Centre located in the municipality of Rio de Janeiro. After medical release, one of them continued the rehabilitation treatment at the same location by means of transportation offered by the city of Nova Friburgo. The other received physiotherapy assistance at home offered by a volunteer. These are two cases of severe injuries with the need for rehabilitation monitoring for a long period, and they offer an empirical basis to reflect on the rehabilitation centres versus Community-based Rehabilitation (CBR).

To Almeida and Campos (2002) [28] the model based on rehabilitation centres losses in capacity to offer wide coverage and focus on the disease or disorder instead of the person. The patient that continued the treatment at the rehabilitation centre declared that the distance and insistent demands to reach technical objectives for recovery were working like barriers, while the one that was assisted at home with less technological resources, but focusing on participation, reached better results. Contrary to the first, the same went back to work and was independent for the instrumental activities of daily life with few adaptations.

Technology does not, necessarily, solve the rehabilitation problem. Well-equipped rehabilitation centres may contribute to functional recovery, but should not be seen as the only choice for rehabilitation assistance. If the treatment is not succeeding, it is necessary to identify and consider with the patient the barriers that are being imposed.

It is possible to affirm that not all of those that needed rehabilitation services effectively searched for it. The increase in the proportion of people with traumatic injuries and the examples of interviewed victims indicated that an episode of repressed demand occurred since the disaster created different rehabilitation needs directly and indirectly. Gelberg et al. (2000) [29] uses the term “competing needs” to refer to the problems of life that prevent people from searching the healthcare system even if they need it, and showing that vulnerable populations are more susceptible to delay the search. In the present case study, the emotional shock, loss of material goods, documents, search for a new residence, represented new needs that competed with the rehabilitation needs.

To access the rehabilitation service, users of SUS as well as private plans, need a medical referral. In this sense, by proposing a model to study the access and use of health services, Dutton (1986) [30] distinguishes the use of services with control predominantly determined by the user, from those in which the physicians control the entry. Considering this model, the rehabilitation services are placed second Thus, it is possible that the lack of medical referrals for the victims of traumatic injuries or diseases indicated for rehabilitation has contributed for the establishment of a situation in which the needs for physical rehabilitation after the disaster were invisible.

The first limitation identified was regarding the quality of hospital records. Many of the hospital records, especially those of the week after the disaster, were incomplete. Thus, the only information we could work with was about the occurrence of traumatic injuries, and it was not possible to associate it with the place of residence or other variables. Some records were torn or wet, which makes us believe that the actual number of calls in the week following the disaster was slightly higher than the records showed.

The second limitation was the fieldwork and interviews performed almost 5 years after the disaster. It may have a bias on the memory aspect. At the same time, one may consider that such a notable event would persist longer, being remembered by those that experienced it.

## Conclusion

Although the disaster has generated new cases of people with traumatic injuries and stress at the same time, leading to an increase in rehabilitation needs, not every need was translated into demand. Some barriers have been identified such as: competing needs (cleaning the house, helping someone, retrieving documents); distance from the rehabilitation centre, unawareness of the rehabilitation services, and possible lack of medical referral. At the same time, those who were treating chronic or less serious conditions were also captured by competing needs and the result was not a large increase in the demand for rehabilitation services. Therefore, it is highly recommended that the health service conduct an active search for people at risk of developing disabilities due to injuries caused by the disaster and avoid the underuse of the health services.

The present study showed that the participation of the physical rehabilitation sector in the recovery stage of the disaster was minimal. Rehabilitation is a slow process and thus it traverses the daily issues. In disaster situations, it is also traversed by new needs that come about in life, from economic, social and also emotional areas. Health services could not stop in immediate response and disease control.

The present study shows that the outpatient rehabilitation needs after a disaster do not generally appear immediately after it. There is an interval of at least 3 months, considering periods of post-surgery recovery and immobilization. Therefore, there is a period for outpatient services to reorganize the assistance and make vacancies available to victims with physical rehabilitation needs after the disaster. In some cases, it will be necessary to bend the schedule, prioritising cases that need more prompt diagnosis and therapeutics, without abandoning patients being monitored.

## Data Availability

The datasets used and/or analysed during the current study are available from the corresponding author on reasonable request.

## Abbreviations

CBR: Community-based Rehabilitation
PHS: Private Health Service
SUS: Unified Health System
